# Feeding Behavior-Related Toxicity due to *Nandina domestica* in Cedar Waxwings (*Bombycilla cedrorum*)

**DOI:** 10.4061/2010/818159

**Published:** 2010-12-09

**Authors:** Moges Woldemeskel, Eloise L. Styer

**Affiliations:** Tifton Veterinary Diagnostic and Investigational Laboratory, Department of Pathology, College of Veterinary Medicine, The University of Georgia, 43 Brighton Road, Tifton, GA 31793, USA

## Abstract

Dozens of Cedar Waxwings were found dead in Thomas County, Georgia, USA, in April 2009. Five of these were examined grossly and microscopically. Grossly, all the examined birds had pulmonary, mediastinal, and tracheal hemorrhages. Microscopically, several tissues and organs were diffusely congested and hemorrhagic. Congestion and hemorrhage were marked in the lungs. Intact and partly digested berries of *Nandina domestica* Thunb. were the only ingesta found in the gastrointestinal tract of these birds. Due to their voracious feeding behavior, the birds had eaten toxic doses of *N. domestica* berries. *N. domestica* contains cyanide and is one of the few berries readily available at this time of the year in the region. The gross and microscopic findings are consistent with lesions associated with cyanide toxicity. This paper for the first time documents toxicity associated with *N. domestica* in Cedar Waxwings.

## 1. Introduction

The Cedar Waxwing (*Bombycilla cedrorum*, formerly *Ampelis cedrorum*) is a member of the family Bombycillidae. It breeds in North America, principally southern half of Canada, and the northern half of the United States [1]. Its winter range includes the United States, Mexico, and Central America as far south as Panama, and the Caribbean region [1, 2]. During winter and spring, when berry supplies are low or out of season, the Cedar Waxwings migrate in huge numbers out of the northern United States and southern Canada into most of the south western and south eastern United States. Cedar Waxwings are opportunistic feeders and move in a nomadic, unpredictable migration following the food supply [3]. During winter they eat fruit almost exclusively [1, 2] and switch to eating mostly insects in summer [1]. Diet analysis from eastern US over 65 years showed that fruit constituted 84% of their annual diet [2]. *N. domestica* forms an excellent backdrop for perennials that disappear in winter. Clusters of the bright red berries of *N. domestica* last for months on each plant, attracting hungry birds whose food is in short supply during this time of the year and into late spring [4]. Cedar Waxwings are voracious feeders, often eating until they can eat no more [5]. They may become intoxicated and die from eating large quantities of overripe fruit [2, 3]. Toxicity associated with *N. domestica* is not previously reported in Cedar Waxwings. 

## 2. Materials and Methods

Dozens of Cedar Waxwings were found dead in Thomas County, Georgia, USA, in April 2009. Five of these were necropsied at Tifton Veterinary Diagnostic and Investigational Laboratory of The University of Georgia, College of Veterinary Medicine. The birds were examined grossly and microscopically. For microscopic examination, tissue samples were collected at postmortem and fixed in 10% buffered formalin, processed for routine histopathology, sectioned at 5 *μ*m, stained with hematoxylin-eosin (H&E), and examined by light microscopy. 

## 3. Results

**Figure 1:**
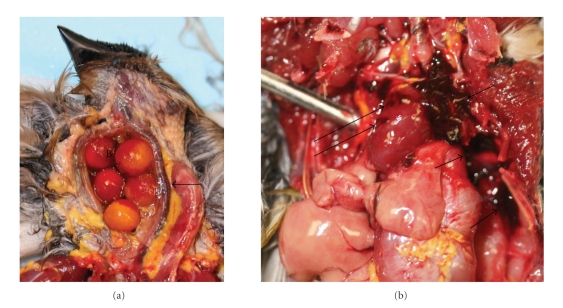
Toxicity of *Nandina domestica* in Cedar Waxwings. (a). Photograph showing berries of *N. domestica* (B) in a crop of dead Cedar Waxwing. The arrow shows congested and hemorrhagic trachea. (b). Photograph showing mediastinal and pulmonary (short arrows) and cardiac hemorrhages (long arrows).

**Figure 2:**
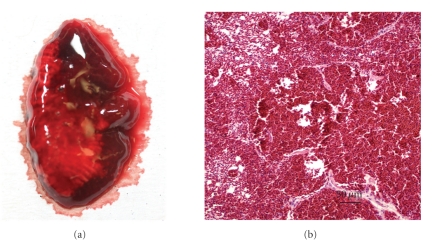
Toxicity of *Nandina domestica* in Cedar Waxwings. (a). Photograph showing pulmonary edema, congestion and hemorrhage. (b). Photomicrograph showing diffuse severe pulmonary congestion and hemorrhage. H&E stain. Bar: 50 *μ*m.

All the examined birds had similar findings on gross and microscopic examinations. Grossly, intact berries of *N. domestica* variably filled the crop (Figure 1(a)). Ample amounts of partly digested berries also filled diffusely orange-stained ventricular lumens. A few intact berries were found in the proventriculus. There was hemorrhage in the lungs, heart, trachea, and thoraco-abdominal cavity (Figures 1(a), 1(b), and 2(a)). 

**Figure 3:**
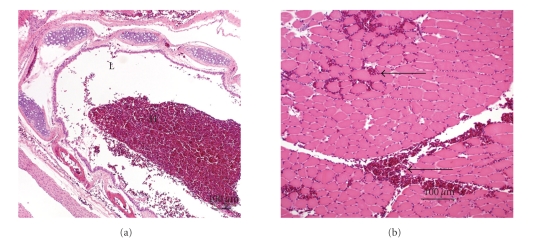
Toxicity of *Nandina domestica* in Cedar Waxwings. (a). Photomicrograph showing hemorrhage (H) in the tracheal lumen (L). H&E stain. Bar: 100 *μ*m. (b). Photomicrograph showing multifocal hemorrhage in skeletal muscle (arrows). H&E stain. Bar: 100 *μ*m.

Microscopically, the lungs, liver, kidney, proventriculus, ventriculus, uvea of the eye, heart, the meninges, and brain were diffusely congested. The hemorrhage and congestion were marked in the lungs (Figure 2(b)). The tracheal lumen (Figure 3(a)) and pulmonary air capillaries were filled with hemorrhage. Multifocally, there was also hemorrhage within the skeletal muscles (Figure 3(b)). The findings in the other examined tissues were unremarkable. 

## 4. Discussion

During winter, the Cedar Waxwings are concentrated in southeastern coastal plains of the USA. They are highly vagile, moving among crops of fruits, including those of ornamental trees and shrubs in suburban areas [6]. Destruction of cultivated fruit is an index to the natural feeding habits of the bird, wild fruits being decidedly favored [5]. In winter, their diets are almost exclusively fruits. At this time of the year, the birds have relied increasingly on crops of ornamental fruits planted in the urban areas in recent years. Ornamental fruiting plants and alien invasives may have shifted distributions of the birds and caused regional population increases [2, 7].* Nandina domestica* is a native of China and Japan. The species and its dwarf varieties are popular landscape items. The plant has naturalized and invaded habitats in southeastern and other areas of the USA. The bright berries are beloved by birds and attract Cedar Waxwings, mockingbirds, and robins [6].

The appetite of the Cedarbird is of so extraordinary nature as to prompt it to devour every fruit or berry that comes in its way [5]. Cedar Waxwings eat bulky fruits that contain easily assimilated simple sugars of glucose and fructose [8]. They can store ingested fruits in a distensible portion of their esophagus, which is likely important in maximizing the amount of fruit ingested per foraging bout that their gizzards and intestines can process at any time [9]. On occasion, they eat fruit that is overripe in such quantities that they become intoxicated [3]. In this manner, they gorge themselves to such excess as sometimes to be unable to fly and suffer themselves to be taken by hand [5]. Intact and partly digested berries of *N. domestica* were the sole contents of the gastrointestinal tract of the examined birds. This indicates that the birds had eaten toxic doses of *N. domestica *berries, one of the few fleshy fruits available in winter and spring in south Georgia. 

*Nandina domestica *berries contain cyanide and other alkaloids [7, 10]. For most cultivars of *N. domestica*, cyanogenesis is the most important intoxication factor [10]. Cyanide glycosides are substances present in many plants that can produce highly toxic hydrogen cyanide (HCN). At least 2000 plant species are known to contain cyanide glycosides with the potential to produce HCN poisoning. Generally, most parts of the plants contain cyanogenic glycocides, the young rapidly growing portion of the plant and the seeds containing the highest concentration. At least 55 cyanogenic glycosides are known to occur in plants, many being synthesized from aminoacids as part of normal plant metabolism. Frost and drought conditions may increase cyanogenesis in some plant species. Cool moist growing conditions enhance the conversion of nitrate to aminoacids and cyanogenic glycosides instead of plant protein [7]. Presumably, similar weather conditions during late winter and early spring in the study area might have favored increased cyanogenesis in *N. domestica.*

Cyanogenic plants represent a problem for various range of animals and wildlife, primarily among species that eat rapidly [11]. The gastrointestinal tract of the examined birds solely contained berries of *N. domestica*. Because of their voracious feeding behavior, the birds have eaten toxic doses of *N. domestica* berries for which cyanogenesis is the most important intoxication factor [10]. Tissue cyanide levels were not measured in these birds since cyanide is rapidly lost from animal tissues unless specimens are collected within a few hours of death and frozen for chemical analysis [7]. 

Hydrogen cyanide is highly poisonous to all animals. Sudden death is often the only presenting sign of acute cyanide poisoning [7]. Although there are marked differences in toxicity of cyanide among species of birds, progression of signs of toxicity to death in birds is generally similar to those reported in mammals [12]. A high rate of cyanide absorption is critical for acute toxicity. If a lethal dose is absorbed, death usually follows within minutes to one hour [13]. Cyanide is a mitochondrial toxin that impairs cellular respiration causing morbidity or mortality within a short time [7, 14]. It is predominantly a neurotoxin, and its toxicity is mediated through inhibition of cytochrome oxidase, an end-chain enzyme of mitochondrial respiration. Cyanide's actions are complex and cannot be attributed solely to deprivation of cellular oxygen. Recent mechanistic studies show that cyanide inhibits multiple enzymes and alters several vital intracellular processes, which lead to a cascade of toxic events [14]. 

In cyanide poisoning, hemorrhages occur commonly in the heart, lungs, and various other organs of affected animals. The characteristic cherry red venous blood seen in acute cyanide poisoning results from the failure of the oxygen-saturated hemoglobin to release oxygen to the tissues because the enzyme cytochrome oxidase is inhibited by cyanide [7]. The gross and microscopic findings in the examined birds are consistent with lesions associated with cyanide toxicity. In concurrence with the finding in the present report, birds that die from cyanide chemical toxicosis have bright red, oxygenated blood, and their tissues or organs, particularly the lungs, may appear congested with blood, hemorrhagic, and edematous [15]. This paper for the first time documents toxicity associated with *N. domestica* in Cedar Waxwings. The berries of *N. domestica* are beloved by other birds such as robins and mockingbirds [6], indicating the potential toxicity to these birds if toxic doses are consumed during feed unavailability.
